# Supplementary material for: Postoperative, but not preoperative, central corneal thickness correlates with the postoperative visual outcomes of Descemet membrane endothelial keratoplasty
Source: PLoS One. 2023 Mar 3;18(3):e0282594. doi: 10.1371/journal.pone.0282594 (PMC9983850; doi:10.1371/journal.pone.0282594)
Supplement: S1 Table — (DOCX) [file pone.0282594.s001.docx]

**Supplementary Table S1.**

Evolution of CCT and BSCVA after surgery

|  | **CCT, µm** | **BSCVA, logMAR** |
| --- | --- | --- |
| Day 8 | 695±110 (441–1221) | 0.78±0.43 (1.7–0) |
| Day 15 | 611±77 (481–857) | 0.5±0.4 (2–-0.1) |
| 1 month | 556±53 (445–775) | 0.28±0.23 (1.3–-0.1) |
| 3 months | 539±52 (415–795) | 0.16±0.16 (0.8–-0.1) |
| 6 months | 538±50 (419–837) | 0.1±0.15 (1–-0.1) |
| 12 months | 538±41 (440–736) | 0.08±0.14 (1–-0.2) |

The data are expressed as mean±standard deviation (range).

BSCVA, Best spectacle-corrected visual acuity; CCT, Central corneal thickness.
