# Supplementary material for: Postoperative, but not preoperative, central corneal thickness correlates with the postoperative visual outcomes of Descemet membrane endothelial keratoplasty
Source: PLoS One. 2023 Mar 3;18(3):e0282594. doi: 10.1371/journal.pone.0282594 (PMC9983850; doi:10.1371/journal.pone.0282594)
Supplement: S2 Table — (DOCX) [file pone.0282594.s002.docx]

# Supplementary Table S2.

Correlations between preoperative and postoperative CCT

|  | **Correlation coefficient**  **r (95% CI)*** | **p value** |
| --- | --- | --- |
| Day 8 | 0.12 (-0.08; 0.31) | 0.999 |
| Day 15 | 0.11 (-0.09; 0.29) | 0.946 |
| 1 month | 0.19 (0.01; 0.36) | 0.217 |
| 3 months | 0.18 (0.01; 0.35) | 0.258 |
| 6 months | 0.16 (-0.01; 0.33) | 0.406 |
| 12 months | 0.21 (0.03; 0.37) | 0.117 |

* Pearson’s correlation test followed by Bonferroni correction.

CCT, central corneal thickness; CI, confidence interval.
