# Supplementary material for: Postoperative, but not preoperative, central corneal thickness correlates with the postoperative visual outcomes of Descemet membrane endothelial keratoplasty
Source: PLoS One. 2023 Mar 3;18(3):e0282594. doi: 10.1371/journal.pone.0282594 (PMC9983850; doi:10.1371/journal.pone.0282594)
Supplement: S3 Table — (DOCX) [file pone.0282594.s003.docx]

# Supplementary Table S3.

Comparison of eyes with low preoperative CCT (<625 µm) and high preoperative CCT (≥625 µm) in terms of postoperative CCT

|  | **Low preoperative CCT (<625 µm)**  ***n*=70** | **High preoperative CCT (≥625 µm)**  ***n*=54** | **p value*** |
| --- | --- | --- | --- |
| Day 8 | 683±128 (441–1221) | 710±80 (580–1032) | 0.999 |
| Day 15 | 603±80 (481–857) | 621±73 (489–840) | 0.999 |
| 1 month | 548±55 (475–775) | 566±49 (445–755) | 0.393 |
| 3 months | 531±55 (415–795) | 549±48 (460–773) | 0.351 |
| 6 months | 533±54 (419–837) | 545±42 (464–711) | 0.942 |
| 12 months | 534±44 (440–736) | 544±37 (464–690) | 0.966 |

The data are expressed as mean±standard deviation.

*The subgroups were compared by Student’s t–test, followed by Bonferroni correction.

CCT, central corneal thickness.
